# Impact of Label Restriction on Checkpoint-Inhibitor Use in Bladder Cancer and Changes in Mortality

**DOI:** 10.1093/jncics/pkac050

**Published:** 2022-07-09

**Authors:** Daniel T Vader, Ravi B Parikh, Haojie Li, Kentaro Imai, Rebecca A Hubbard, Ronac Mamtani

**Affiliations:** Department of Biostatistics, Epidemiology and Informatics, University of Pennsylvania, Philadelphia, PA, USA; Department of Medical Ethics and Health Policy, University of Pennsylvania, Philadelphia, PA, USA; Division of Hematology and Oncology, Department of Medicine, University of Pennsylvania, Philadelphia, PA, USA; Merck & Co, Inc, Kenilworth, NJ, USA; Merck & Co, Inc, Kenilworth, NJ, USA; Department of Biostatistics, Epidemiology and Informatics, University of Pennsylvania, Philadelphia, PA, USA; Division of Hematology and Oncology, Department of Medicine, University of Pennsylvania, Philadelphia, PA, USA

## Abstract

In 2018, the US Food and Drug Administration (FDA) limited the indication for immune checkpoint inhibitors (ICI) in metastatic bladder cancer to patients with programmed cell death protein ligand-1 (PD-L1)–positive tumors. The impact of the label change on survival outcomes remains unknown. We conducted a controlled interrupted time series analysis using a nationwide electronic health record–derived oncology dataset. We used Cox regression to compare mortality in the post- vs prelabel change periods among affected (initiators of ICI or carboplatin-based chemotherapy) vs unaffected (initiators of cisplatin-based chemotherapy) patients. The use of ICI, carboplatin, and cisplatin was similar pre- and postlabel change, but PD-L1 testing increased postlabel change. In adjusted models, survival did not differ after the FDA label change policy compared with prior to the label change in any of the groups. The FDA label restriction on immunotherapy was associated with increased PD-L1 testing but not with changes in treatment patterns or mortality among patients with metastatic bladder cancer.

Cisplatin-based chemotherapy is the standard of care for patients with metastatic bladder cancer. However, more than half of all patients with bladder cancer are not eligible for cisplatin-based chemotherapy because of comorbidity (1). Carboplatin-based chemotherapy is often used in this setting. In April 2017, 2 immune checkpoint inhibitors (ICIs), atezolizumab and pembrolizumab, received accelerated approval for the first-line treatment of patients who are cisplatin ineligible. This approval was based on results from 2 single-arm phase II studies, irrespective of programmed cell death protein ligand-1 (PD-L1) expression level (2,3). In June 2018, the US Food and Drug Administration (FDA) and European Medicines Agency restricted this indication to cisplatin-ineligible patients with PD-L1–positive tumors (4,5). This decision was based on early review of data from confirmatory phase III trials that suggested decreased overall survival in patients with PD-L1–negative tumors when treated with ICI monotherapy compared with platinum-based chemotherapy (6,7). Our prior data suggested that the label change led to short-term decreases in ICI use, but its impact on survival outcomes among patients with metastatic bladder cancer is unknown (8).

We conducted a controlled interrupted time series analysis to investigate the impact of the FDA label change on mortality using data from the US nationwide electronic health record–derived Flatiron Health database (9). The study sample included patients from 280 cancer clinics who started first-line therapy for metastatic bladder cancer between April 1, 2017, and March 1, 2020. The University of Pennsylvania institutional review board determined that this study met eligibility for institutional review board exemption with a waiver of informed consent owing to the use of deidentified retrospective data.

Using multivariable Cox regression, we compared overall survival between the prelabel change period (April 1, 2017, to May 17, 2018) and the postlabel change period (June 20, 2018, to March 1, 2020) among patients affected by the label change (initiators of ICI or carboplatin-based chemotherapy) and those unaffected by the label change (initiators of cisplatin-based chemotherapy). We excluded patients initiating therapy during a 30-day washout period, defined as the time period between the initial FDA safety alert (May 18, 2018) and the official FDA label change (June 19, 2018). The Cox model included terms for label change period, treatment group, period by treatment interaction, and baseline demographic (gender, race and ethnicity, age) and clinical (smoking status, primary tumor site, body mass index, creatinine clearance, and calendar day of diagnosis) covariables. Using this model, we estimated mortality hazard ratios comparing the post- and prelabel change periods within each treatment group, and we tested for interaction between treatment group and time period terms. We report 95% confidence intervals and 2-sided *P* values and set alpha a priori to .05.

The proportional hazards assumption was assessed using Schoenfeld residuals. We accounted for missing covariate data using multiple imputation via chained equations and accounted for practice-level clustering using robust standard errors. Follow-up continued until death or date of last structured activity in the electronic health record prior to date of data extraction (June 1, 2021).

Our sample included 829 patients who initiated first-line therapy in the prelabel change period (582 [70.2%] in the label change affected group and 247 [29.8%] in the label change unaffected group) and 1184 in the postlabel change period (849 [71.7%] in the label change affected group and 336 [28.4%] in the label change unaffected group). Use of ICI, carboplatin-based chemotherapy, and cisplatin-based chemotherapy was similar across time periods, whereas PD-L1 testing increased from 6.6% to 28.1% (Figure 1;Supplementary Table 1, available online). Kaplan-Meier survival estimates were similar between the pre- and postlabel change periods, and the adjusted model identified no significant difference in the overall survival hazard between the 2 time periods in either the label change affected or unaffected group (Figure 2).

**Figure 1.**
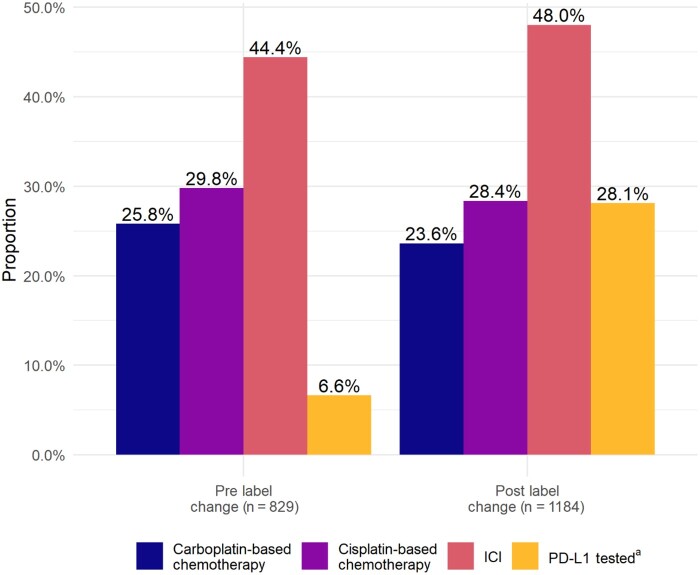
First-line treatment and testing patterns by label change period. ^a^PD-L1 status was determined using recorded results from Dako 22C3 assays, Ventana SP142 assays, and written lab interpretations (positive or negative). Patients with results on any of these indicators were classified as tested. We classified patients as PD-L1 positive if they had a combined positive score of ≥10%, percent staining ≥5%, or positive lab within 30 days after or any time prior to treatment initiation. Patients were classified as negative if they had at least 1 PD-L1 indicator on record (from any of the 3 fields) and no positive tests. ICI = immune checkpoint inhibitors; PD-L1 = programmed cell death protein ligand-1.

**Figure 2.**
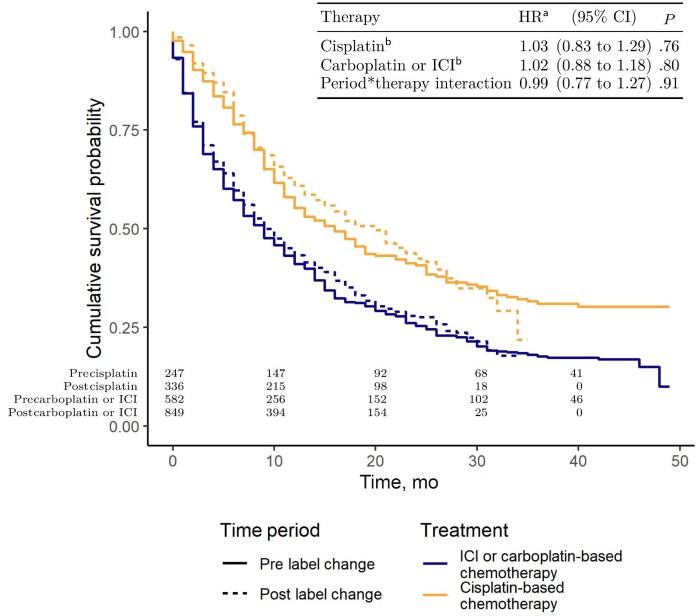
Cumulative survival probabilities by treatment and label change period and adjusted hazard ratios. ^a^Adjusted for sex (female or male), smoking status (ever or never), race and ethnicity (Black, Latinx, White, other), primary site (bladder or nonbladder), age, body mass index, creatinine clearance, and calendar day of diagnosis. ^b^Hazard ratio comparing post- vs prelabel change within treatment group. CI = confidence interval; HR = hazard ratio; ICI = immune checkpoint inhibitors.

The FDA label restriction on ICIs to patients with PD-L1–positive bladder cancer was associated with a modest increase in PD-L1 testing but was not associated with long-term changes in treatment selection or mortality. Potential explanations for these findings include limited adherence to the label revision by clinicians and/or limited effectiveness of PD-L1–guided treatment selection. Limitations of this analysis include inability to stratify analyses by PD-L1 status because of the overall low sample size of patients receiving testing prior to and after the label change. Further, we were unable to account for differences in the treatment environment (eg, the introduction of maintenance immunotherapy) that may have differentially impacted survival in label change affected vs unaffected groups. However, the potential effect of maintenance immunotherapy on our results is likely minimal because only a limited subset of patients were eligible. Finally, we could not assess changes in the proportion of ICI initiators who were ineligible for any platinum chemotherapy (cisplatin and carboplatin) between the 2 time periods, as formal criteria used to define this subset are absent, yet these patients remain eligible for ICIs in the postlabel change period regardless of PD-L1 status. For drugs receiving accelerated approval, label change restrictions based on early review of clinical trials may have limited impact on utilization and outcomes in oncology care.

## Funding

This work was supported by Merck & Co.

## Notes

**Role of the funder:** The funder had no role in the design of the study; the collection or analysis of the data; the writing of the manuscript; and the decision to submit the manuscript for publication; but did participate in the interpretation of the data and the critical review of the manuscript.

**Disclosures:** Daniel Vader, Ravi Parikh, and Ronac Mamtani have received personal fees from Flatiron Health. Ravi Parikh has received grant funding from Humana, personal fees and equity from GNS Healthcare Inc and Onc.AI, and personal fees from the Cancer Study Group and Nanology outside the submitted work. Haojie Li and Kentaro Imai report employment at Merck & Co, Inc. Rebecca Hubbard has received grant funding (to institution) from Pfizer, Johnson & Johnson, and Merck. Ronac Mamtani has received grant funding (to institution) from Merck, and personal fees from BMS, Astellas, and SEAGEN.

**Author contributions:** All authors: conceptualization, writing—review & editing. RH, RM, and DV: methodology. DV: formal analysis and data curation. RM, DV, RH, and RP: original draft, data visualization. RM and HL: project administration.

**Prior presentations:** Vader DT, Parikh RB, Li H, Imai K, Hubbard RA, Mamtani R. Impact of FDA label change on immunotherapy for metastatic urothelial cancer (mUC) and subsequent changes in mortality. San Francisco, CA; American Society of Clinical Oncology Genitourinary Cancers Symposium; 2022.

## Data Availability

All analytic code is available at https://github.com/daniel-vader/mUCC-ICI-label-restriction. The data that support the findings of this study have been originated by Flatiron Health, Inc. These de-identified data may be made available upon request and are subject to a license agreement with Flatiron Health; interested researchers should contact DataAccess@flatiron.com to determine licensing terms.

## Supplementary Material

pkac050_Supplementary_Data
