# Supplementary material for: Impact of Label Restriction on Checkpoint-Inhibitor Use in Bladder Cancer and Changes in Mortality
Source: JNCI Cancer Spectr. 2022 Jul 9;6(4):pkac050. doi: 10.1093/jncics/pkac050 (PMC9364375; doi:10.1093/jncics/pkac050)
Supplement: pkac050_Supplementary_Data [file pkac050_supplementary_data.pdf]

Supplementary Materials

Supplementary Table 1: Baseline characteristics of sample

	Pre-label change		Post-label change		Total	
Category	No.	%	No.	%	No.	%
Gender						
Female	205	24.7%	342	28.9%	547	27.2%
Male	624	75.3%	842	71.1%	1466	72.8%
Missing	0		1		1	
Race/ethnicity						
White	578	76.2%	782	72.7%	1360	74.1%
Black	37	4.9%	48	4.5%	85	4.6%
Latinx	39	5.1%	36	3.3%	75	4.1%
Other	105	13.8%	210	19.5%	315	17.2%
Missing	70		109		179	
Therapy group						
Carboplatin	214	25.8%	280	23.6%	494	24.5%
Cisplatin	247	29.8%	336	28.4%	583	28.9%
Immunotherapy	368	44.4%	569	48.0%	937	46.5%
Primary site						
Bladder	640	77.2%	893	75.4%	1533	76.1%
Renal Pelvis	107	12.9%	164	13.8%	271	13.5%
Ureter	76	9.2%	121	10.2%	197	9.8%
Urethra	6	0.7%	7	0.6%	13	0.6%
History of smoking	614	74.1%	871	73.5%	1485	73.7%
Missing	0		2		2	
Steroids	39	4.7%	48	4.1%	87	4.3%
Opioids	48	5.8%	75	6.3%	123	6.1%
ECOG PS: 2+	155	24.1%	194	20.4%	349	21.9%
Missing	186		232		418	
Insurance						
Commercial	403	48.6%	643	54.3%	1046	51.9%
Medicare	196	23.6%	272	23.0%	468	23.2%
Other/self pay/not specified	210	25.3%	247	20.8%	457	22.7%
Other government insurance	20	2.4%	23	1.9%	43	2.1%
PD-L1 tested[a]	55	6.6%	333	28.1%	388	19.3%
PD-L1 positive[a]	17	32.7%	131	41.9%	148	40.5%
Missing	777		872		1649	
Age (median, IQR)	72.1	(67.0, 80.0)	72.6	(67.0, 81.0)	72.4	(67.0, 81.0)
BMI (median, IQR)	26.8	(22.9, 30.3)	27.3	(23.3, 30.6)	27.1	(23.1, 30.4)
Missing	6		17		23	
Creatinine clearance (median, IQR)	63.5	(42.5, 79,3)	63.3	(41.1, 80.3)	63.4	(41.8, 80.1)
Missing	34		56		90	

[a] PD-L1 status was determined using recorded results from Dako 22C3 assays, Ventana SP142 assays, and written lab interpretations ("positive" or "negative"). Patients with results on any of these indicators were classified as “tested.” We classified patients as PDL/1 positive if they had a combined positive score of ≥10%, percent staining ≥5%, or "positive" lab within 30 days after or any time prior to treatment initiation. Patients were classified as negative if they had at least one PD-L1 indicator on record (from any of the three fields) and no positive tests. ECOG PS, Eastern Cooperative Oncology Group Performance Status; PD-L1, programmed death-ligand 1; IQR, interquartile range
